# Simplifying minimally invasive right hepatectomy

**DOI:** 10.1007/s00464-023-09996-7

**Published:** 2023-04-07

**Authors:** Nora Nevermann, Linda Feldbrügge, Sebastian Knitter, Felix Krenzien, Nathanael Raschzok, Georg Lurje, Wenzel Schöning, Johann Pratschke, Moritz Schmelzle

**Affiliations:** 1 Department of Surgery, Campus Charité-Mitte and Campus Virchow-Klinikum, Charité – Universitätsmedizin Berlin, Corporate Member of Freie Universität Berlin, Humboldt-Universität zu Berlin, and Berlin Institute of Health, Berlin, Germany; 2 Clinician Scientist Program, Berlin Institute of Health (BIH), Anna-Louisa-Karsch-Str. 2, 10178 Berlin, Germany

**Keywords:** Laparoscopic liver surgery, Robotic liver surgery, Hepatectomy, Glissonean approach

## Abstract

**Background:**

Extrahepatic transection of the right hepatic artery and right portal vein before parenchymal dissection is a widely used standard for minimal invasive right hepatectomy. Hereby, hilar dissection represents a technical difficulty. We report our results of a simplified approach in which the hilar dissection is omitted and the line of dissection is defined with ultrasound.

**Methods:**

Patients undergoing minimally invasive right hepatectomy were included. Ultrasound-guided hepatectomy (UGH) was defined by the following main steps: (1) ultrasound-guided definition of the transection line, (2) dissection of the liver parenchyma according to the caudal approach, (3) intraparenchymal transection of the right pedicle and (4) of the right liver vein, respectively. Intra- and postoperative outcomes of UGH were compared to the standard technique. Propensity score matching was performed to adjust for parameters of perioperative risk.

**Results:**

Median operative time was 310 min in the UGH group compared to 338 min in the control group (*p* = 0.013). No differences were observed for Pringle maneuver duration (35 min vs. 25 min; *p* = ns) nor postoperative transaminases levels (*p* = ns). There was a trend toward a lower major complication rate in the UGH group (13 vs. 25%) and a shorter median hospital stay (8 days vs. 10 days); however, both being short of statistical significance (*p* = ns). Bile leak was observed in zero cases of UGH compared to 9 out of 32 cases (28%) for the control group (*p* = 0.020).

**Conclusions:**

UGH appears to be at least comparable to the standard technique in terms of intraoperative and postoperative outcomes. Accordingly, transection of the right hepatic artery and right portal vein prior to the transection phase can be omitted, at least in selected cases. These results need to be confirmed in a prospective and randomized trial.

**Graphical Abstract:**

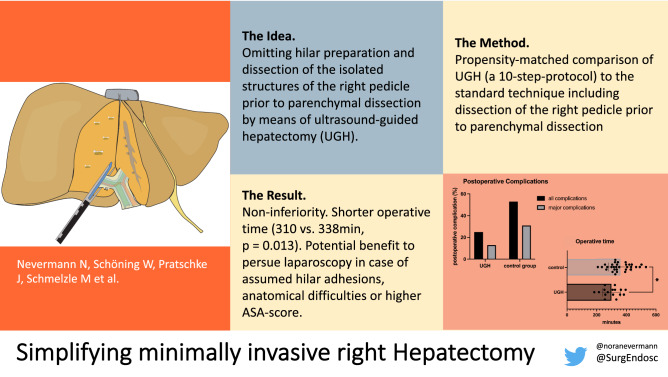

For minimally invasive right hepatectomies, two surgical approaches can be distinguished. Following the definition introduced by Couinaud, the techniques can be classified as intrafascial and extrafascial [1]. The intrafascial approach consists of dissection of the hepatoduodenal ligament and isolation of the right hepatic artery and the right portal vein (and eventually the right bile duct). After individual transection of the structures, the resulting demarcation line visualizes the anatomical Cantlie’s line separating the right and the left liver lobe and parenchymal transection can be performed accordingly. The extrafascial or Glissonean pedicle approach was first described by Couinaud [2] and further developed by Takasaki and others [3]. Hereby, the right hepatic pedicle can be isolated after lowering of the hilar plate respecting the landmarks and gates that allow access to the primary branches of the portal triad [4].

The intrafascial approach allows early vascular control and parenchymal dissection following a demarcated line but bears the risk of injuring the vessels or the bile duct of the left liver as a result of wrong identification or due to anatomical variations. The extrafascial approach on the other hand demands the delicate detachment of the Glissonean pedicle from Lannaec’s capsula, which can be difficult especially after previous operations and adhesions at the hilum. Our study describes another option with omission of hilar preparation. As adopted from living donor hepatectomy, the parenchymal dissection line (Cantlie’s line) can be defined using the hepatic middle vein as the key anatomical landmark, thereby not depending on parenchymal demarcation [5]. Intraoperative ultrasound can repeatedly be performed for intraparenchymal guidance. During the transection phase, the right pedicle (Fig. 1**)** and right liver vein both get exposed and can be dissected safely by vascular staplers.

**Fig. 1:**
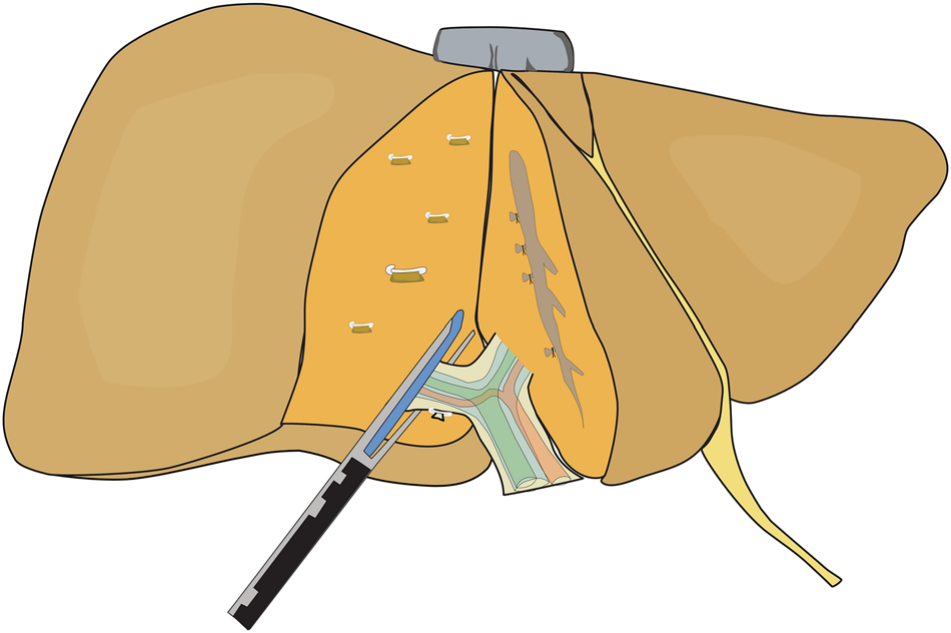
Schema of intraparenchymal pedicle transection after dissection of the liver parenchyma in a caudal approach

We established a 10-step-protocol for the facilitated ultrasound-guided hepatectomy (UGH) with intraparenchymal transection of the right pedicle and right liver vein. Both, extrahepatic isolation of the Glissonean pedicle (classical extrafascial approach) as well as dissection of the hepatoduodenal ligament (intrafascial approach) are thereby avoided. Our study evaluates the feasibility of UGH and compares its intraoperative and postoperative outcome to the established intrafascial approach (control group).

## Materials and methods

### Study design and data collection

As part of a prospective observational study (DRKS00017229), clinical data of all consecutive robotic right hepatectomies between January 1st of 2019, and December 31st of 2020, was collected prospectively at the Department of Surgery, Campus Charité-Mitte and Campus Virchow-Klinikum, Charité- Universitätsmedizin Berlin. Clinical data of all consecutive laparoscopic right hepatectomies performed between January 1st of 2019, and December 31st of 2020, were collected in a retrospective design.

Approval of the Charité institutional review board was obtained (EA4/084/17) and all data was collected, stored, and processed according to the General Data Protection Regulation and local data protection laws. The study was conducted in accordance with the ethical standards of the Helsinki Declaration (1975).

Data on patients’ baseline characteristics, physical status and the underlying pathology were collected as well as parameters of intraoperative and postoperative outcome. Baseline characteristics included age, sex, body mass index (BMI) and the American Society of Anesthesiologists’ Physical Status Classification (ASA score). The IWATE-criteria were included as parameters of surgical complexity [6]. The presence of liver fibrosis was defined as part of the histopathological examination and graded according to the classification of Desmet and Scheuer [7]. Cirrhosis was defined as a fibrosis grade IV. The Child–Pugh Score was used to assess the severity of liver cirrhosis [8].

Cumulative duration of intermittent hilar occlusion (Pringle maneuver) and operative time were recorded as parameters of intraoperative outcome. Length of ICU-stay, length of hospital stay, postoperative level of the transaminases and complication rate at 90 days after surgery were recorded as parameters of postoperative outcome. The level of the transaminases alanine transaminase (ALT) and aspartate transaminase (AST) was measured preoperatively (t0), at 24 h after the surgery (t1) and before discharge (t2). The Clavien-Dindo-Classification was used for severity grading of postoperative complications [9]. Major complications were defined as a Clavien-Dindo-score of ≥ IIIa.

Cases with simultaneous resection of other organs were excluded from the study. Patients were grouped according to the type of surgical technique. The facilitated approach (UGH) was performed following a 10-step-protocol as described below and defined as the study group. Cases with prior hilar preparation and selective ligation of the right hepatic artery and portal vein—according to the extrafascial approach—were defined as the control group. Both groups were compared with regard to baseline characteristics, and intra- and postoperative outcomes.

### Surgical technique

The operation was performed with the patient laid in French position and the surgeon (laparoscopic) or table side surgeon (robotic) standing between the patient’s legs.

For laparoscopic procedures, six trocars were inserted, one umbilical trocar (12 mm diameter), two trocars (12 mm diameter) into the right middle abdomen, two trocars (12 mm diameter) into the median plane of the upper abdomen and one trocar (5 mm diameter) into the left middle abdomen (for external Pringle). For robotic procedures, four robotic trocars (8 mm diameter) were inserted in a linear oblige formation of the right upper abdomen. Three additional trocars were inserted (umbilical position, 12 mm, right middle abdomen, 12 mm, and the left lateral abdomen, 5 mm).

**Fig. 2:**
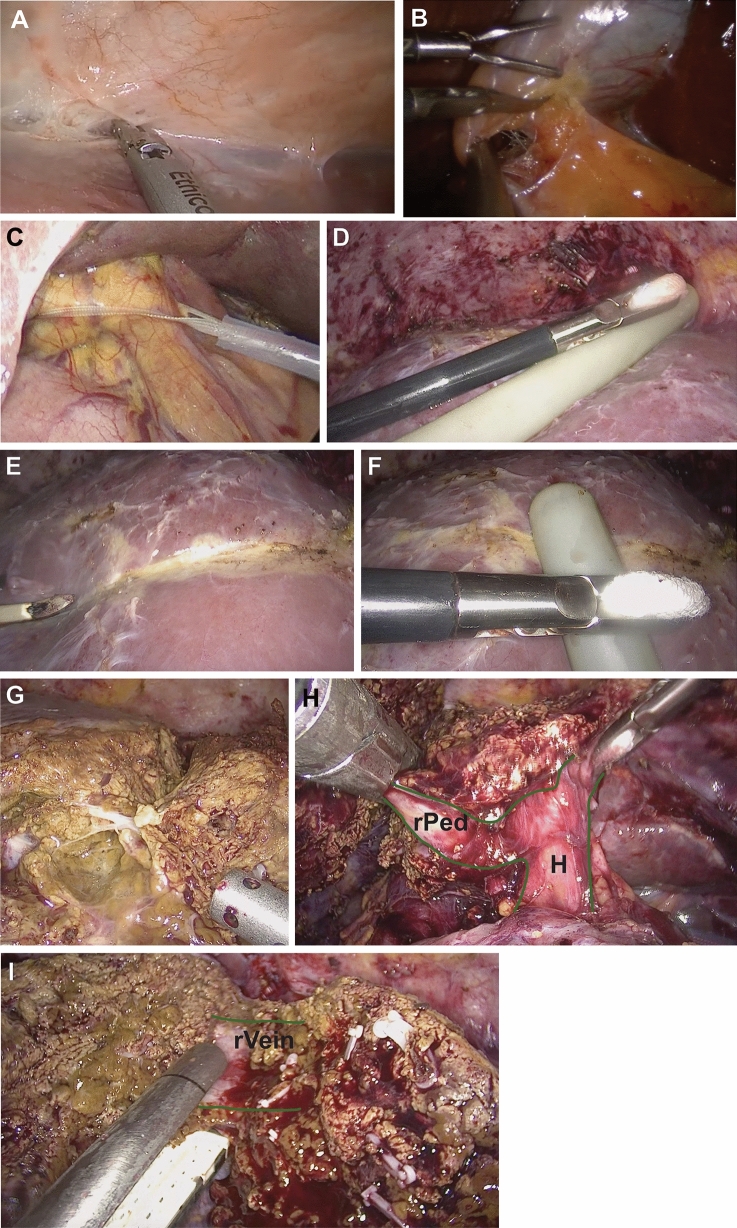
Laparoscopic right hepatectomy with ultrasound-guided (UGH) technique: Demonstration of the 10-step protocol. *H* hepatic hilum, *rPed* right hepatic pedicle, *rVein* right hepatic vein. (**A**) Identification of the grooves adjacent to the right and the middle hepatic vein (step 1), (**B**) cholecystectomy (**C**) Preparation of hepatic inflow occlusion. The hepatoduodenal ligament is encircled with a Mersilene tape (step 3), (**D**–**F**) Ultrasound-guided definition of the parenchymal transection line (step 4). (**G**) Parenchymal transection: Laparoscopic parenchymal transection is performed using ultrasonic shears for surface transection and water jet dissection for deeper transection (step 5). Veins are ligated using polymer clips, (**H**) intraparenchymal stapler dissection of the right hepatic pedicle (step 6). (**I**) stapler dissection or the right liver vein (step 7). Steps 8-10 do not differ from the standard technique and are not depicted in the figure.

UGH was conducted based on a standardized 10-step-protocol consisting of the following steps (Fig. 2**)**: (1). Division of the round and falciform ligaments and identification of the grooves adjacent to the right and the middle hepatic vein. (2). Cholecystectomy. (3). Preparation of hepatic inflow occlusion: The hepatoduodenal ligament is encircled with a Mersilene tape. Intermittent Pringle maneuver is later applied if considered necessary for a maximum duration of 15 min followed by 5 min interruption intervals (4). Ultrasound-guided definition of the parenchymal transection line—the Cantlie line is marked by cauterization. (5). Parenchymal transection: Laparoscopic parenchymal transection is performed using ultrasonic shears (THUNDERBEAT®, Olympus Medical Europa, Hamburg, Germany; HARMONIC‌® HD 1000i, Johnson & Johnson Medical GmbH Ethicon, Norderstedt, Germany) for surface transection and water jet dissection (ERBEJET® 2, Erbe medical; Tübingen, Germany) for deeper transection. Veins are ligated using polymer clips (Lapro-Clip™, Medtronic, Meerbusch, Germany). For robotic resections, the superficial and deep parenchymal dissection is performed using ultrasonic shears (Harmonic Ace ®, Johnson & Johnson Medical GmbH Ethicon, Norderstedt, Germany). For deep tissue dissection, a modified clamp-crush technique is applied for safe identification of relevant structures.

6. Central stapler dissection of the right hepatic pedicle (ECHELON™ + Stapler, reloads: GST60B (60 mm), Johnson & Johnson Medical GmbH Ethicon, Norderstedt, Germany) 7. Stapler dissection of the right hepatic vein (ECHELON™ + Stapler, reloads: GST60B, s. above) 8. Dissection of adhesions of the right lobe with the retroperitoneum and diaphragm. Mobilization of the right liver lobe can also be performed prior to the parenchymal dissection if preferred. 9. Retrieval of the resected tissue: the resected tissue is placed in a retrieval bag and is removed 10. Placement of drainage, control and wound closure.

Standard minimal invasive right hepatectomy (control group) was conducted with selective dissection of the right hepatic artery and right portal vein prior to the parenchymal dissection. The structures were isolated, ligated using polymer clips (Lapro-Clip™, Medtronic, Meerbusch, Germany) and then divided. After the parenchymal dissection, the right bile duct and the right hepatic vein were each dissected with a linear stapler (ECHELON™ + Stapler, reloads: GST60B (60 mm), Johnson & Johnson Medical GmbH Ethicon, Norderstedt, Germany), respectively. We refer to previous publications for technical details [10].

### Perioperative management

Computed tomography (CT) scanning of the chest, abdomen, and pelvis was performed as part of the preoperative staging. If needed, magnetic resonance imaging (MRI) of the liver was performed additionally. For all patients with suspected malignancy, the surgical indication was confirmed by our multidisciplinary tumor board. The choice of the laparoscopic modality and surgical technique was made individually based on an informed decision of the patient and the surgical team. Patient’s consent on the surgical modality, technique and the clinical study was obtained for all cases.

For postoperative surveillance, patients were routinely admitted to the intensive care unit (ICU). An abdominal drain was placed in contact to the parenchymal resection site in all cases and removed 48 h post-surgery given the absence of abnormal secretion.

### Statistical analysis

A propensity score matching was performed to adjust for parameters of perioperative risk. Age, BMI and the IWATE-score (including the parameters tumor size, presence of liver cirrhosis and tumor distance to a major vessel) were thereby defined as confounding variables. A 1:2 matching was performed using the nearest neighbor method, with a caliper set at 0.1. Categorical variables were expressed as frequencies and percentages and were compared using cross tables and a chi-squared test or Fisher’s exact test as appropriate. Continuous variables were expressed as median and interquartile range (IQR), and a Mann–Whitney U test was used for comparison. A *p* value < 0.05 was considered statistically significant. IBM SPSS Statistics version 25 (IBM Corp., Armonk, NY, USA) was used for data analysis.

## Results

Between January 1st of 2019, and December 31st of 2020, ninety-three patients underwent right hepatectomy at the Department of Surgery, Campus Charité-Mitte and Campus Virchow-Klinikum, Charité- Universitätsmedizin Berlin. Of those, twenty-nine cases were performed via open surgery and excluded from analysis. Furthermore, two cases included partial colectomy and were also excluded from analysis. The remaining 62 cases of minimal invasive right hepatectomy were included in our study. Of those, sixteen cases were performed using UGH.

Forty-two out of 62 cases were performed via robotic surgery (68%) and 20 cases via laparoscopic surgery (32%). Within the UGH group (*n* = 16), 3 cases (19%) were robotic resections.

### Baseline characteristics

Distribution of baseline characteristics including median age, BMI, IWATE-score and ASA-score are shown in Table 1. Malignant entities were the most common indication for liver resections (87%) with colorectal liver metastases (40%), hepatocellular carcinoma (23%) and intrahepatic cholangiocarcinoma (16%) representing the most frequent entities. After 2:1 propensity score matching, no significant difference was seen between the UGH and the control group for baseline characteristics, comorbidities and distribution of underlying disease (Table 1) and 16 cases were included to the study group and 32 matched cases to the control group.

**Table 1:** Baseline characteristics prior to and after propensity score matching.

	Prior to matching	After propensity score matching		
	All	UGH	Control group	*p*
	*n* = 62	*n* = 16	*n* = 32	
Age (years)	60 (16)	65 (17)	65 (15)	0.780
Sex (female)	25 (40%)	5 (31%)	11 (34%)	1.000
BMI (kg/m2)	25 (6)	25 (7)	25 (6)	0.624
ASA ≥ 3	23 (37%)	4 (25%)	10 (31%)	0.665
Liver cirrhosis	4 (7%)	1 (6%)	1 (6%)	1.000
Alcohol	1 (2%)	1 (6%)	0	0.310
Smoking	13 (21%)	6 (38%)	2 (13%)	0.220
Diabetes	4 (7%)	1 (6%)	1 (6%)	1.000
Pathology				
CRLM	25 (40%)	5 (31%)	15 (47%)	0.363
HCC	14 (23%)	4 (25%)	8 (25%)	1.000
ICCA	9 (15%)	5 (15%)	3 (19%)	1.000
Other malig	5 (8%)	2 (13%)	2 (6%)	1.000
FNH	0	0	0	
Adenoma	4 (7%)	2 (13%)	1 (3%)	0.106
Hemangioma	2 (3%)	0	0	
Other benign	0	0	0	
IWATE score	10 (1)	10 (2)	10 (2)	0.937
Neoadjuvant systemic therapy	13 (21%)	2 (13%)	5 (16%)	0.700

Values are expressed as percentage or median with interquartile range

### Intraoperative outcome

**Fig. 3:**
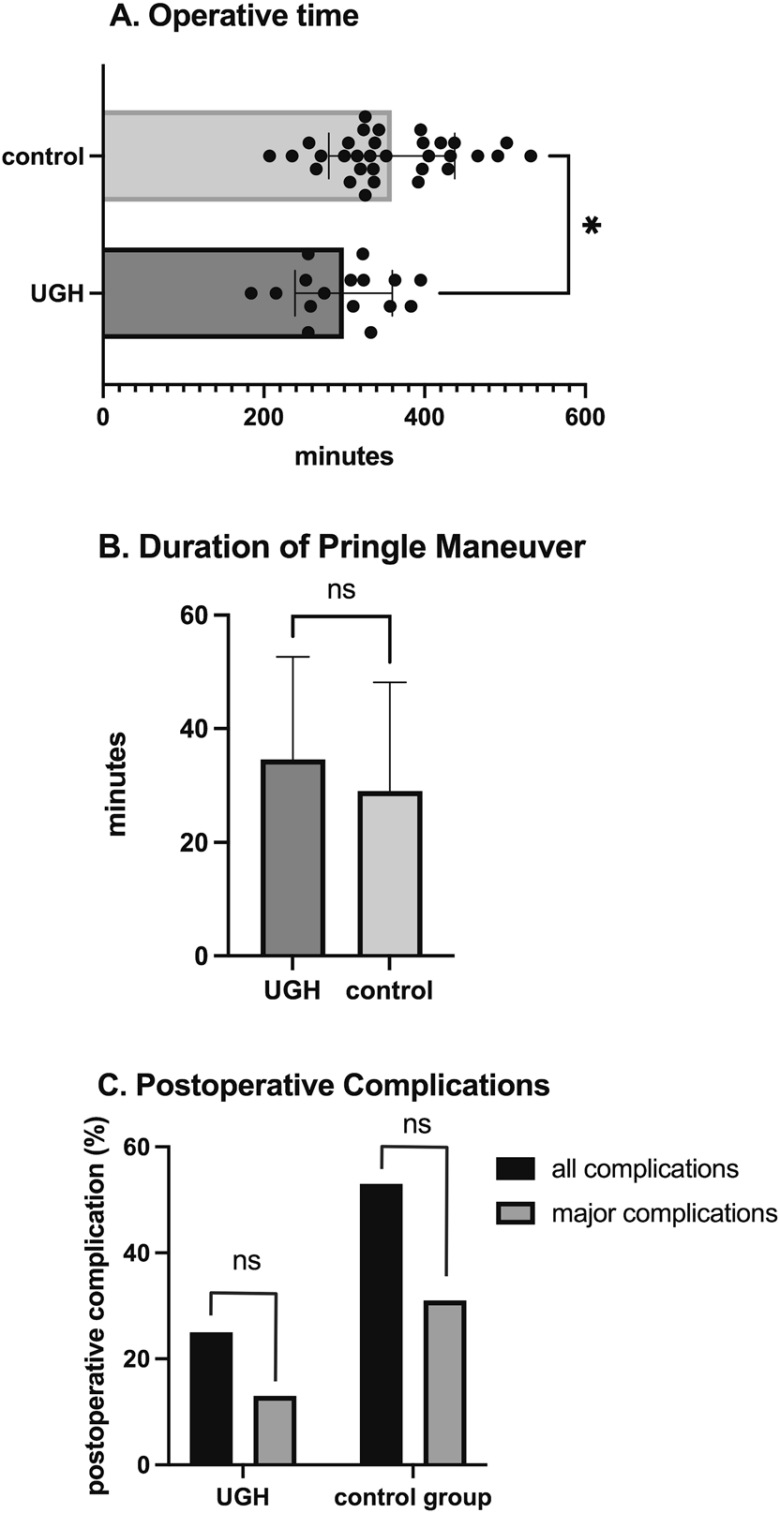
Intra- and postoperative outcomes for right hepatectomy comparing the facilitated approach (UGH) to the standard technique (control group)

Median operative time was 310 min (IQR: 96 min) for the UGH group compared to 338 min (IQR: 107 min) for the control group (*p* = 0.013), Figure 3A.

Pringle maneuver was used in 14 out of 16 cases (88%) of UGH cases compared to 16 out of 32 (50%) within the control group (*p* = 0.027). Pringle maneuver was applied for 35 min (median, IQR: 30 min) within the UGH group compared to 25 min within the control group (IQR: 30 min), *p* = 0.417, as visualized in Fig. 3B.

### Postoperative outcome

Major postoperative complications (Clavien Dindo ≥ IIIa) were recorded in 2 out of 16 cases (13%) within the UGH group compared to 8 out of 32 for the control group (25%), (*p* = 0.589), Fig. 3C.

No difference was observed for AST and ALT levels on t0 (*p* = 0.669 and 0.638), t1 (*p* = 0.406 and 0.922) and t2 (*p* = 0.0381 and 0.320) between the UGH and control group.

Bile leak was observed in zero cases of the UGH group compared to 9 out of 32 cases (28%) for the control group, *p* = 0.020. Within the control group, 6 out of 8 complications were due to a relevant bile leak requiring ERCP or percutaneous drainage. Zero bile leaks requiring intervention were recorded within the UGH group (*p* = 0.091).

Median hospital stay was 8 days (IQR 4) for the UGH group compared to 10 days (IQR 7), *p* = 0.162.

Mean tumor size was 73 mm (SD: 57 mm) and 69 mm (SD: 37 mm) for the UGH and control group (*p* = 0.734) with 5 (39%) and 11 (31%) cases presenting a tumor size above 10 cm. R0 resection rate was 81% (13/16) for the UGH group compared to 66% (21/32) for the control group, *p* = 0.539.

## Discussion

We here report on a simplified technique for minimally invasive right hepatectomy that does neither require dissection of the hepatoduodenal ligament nor pedicle isolation according to an extrafascial Glissonean approach. Our results suggest at least comparable intra- and postoperative outcomes for the UGH, when compared to the standard approach. In our opinion, UGH should be considered as an applicable alternative for minimally invasive right hepatectomy, at least in selected patients with expected difficult hilar preparation. Prior surgery, inflammatory processes or anatomical variation might be indications for choosing UGH as well as patients with ASA-score ≥ 2 who profit particularly from short operative times.

The ILLS Laparoscopic Liver Surgery Fellow Skills Curriculum defines the dissection of the hepatoduodenal ligament among the five most difficult surgical substeps out of 22 defined substeps with a difficulty level of 3.3 (scale from 1 to 5) [11].

Yamamoto describes the extrafascial techniques as a “simple and versatile application procedure” for anatomical hepatectomy [1], but the above mentioned Skills Curriculum still accords a difficulty grading of 3.1 to the Glissonean approach. By replacing either technique with an intraparenchymal transection of the right pedicle and right vein we presume a relevant facilitation of the procedure. This advantage might be enhanced for cases in which hilar lymphadenectomy is not required, i.e., hepatectomies for hepatocellular carcinoma or colorectal metastases.

Parenchymal dissection following a demarcation line is not possible within the UGH, which is a potential disadvantage. For living donor hepatectomy, however, parenchymal dissection prior to ligation of the right or left hepatic artery or portal vein branch has been established over the last 20 years [5, 12]. In the absence of a demarcation line, sufficient guidance is granted using the middle hepatic vein with repeated intraoperative ultrasound controls. Blood inflow control can safely be achieved with intermittent Pringle maneuver. Besides ischemic demarcation, ICG-fluorescence can be used for segmental mapping and to determine a precise resection line on the hepatic surface and also on the intersegmental parenchymal plane [13]. We offer a newly technique that aims to reduces operative risk by avoiding hilar preparation and compare the approach to a standard technique. ICG-fluorescence is not used routinely in European centers but can be valuable addition to laparoscopic liver surgery.

Our results show a significantly reduced operative time for the UGH compared to the control group (*p* = 0.013). This finding supports the assumption that an intraparenchymal pedicle transection can simplify the surgical procedure. Landmark- and ultrasound-guided parenchymal dissection with no prior hilar preparation can reduce the procedure’s duration. This could also be due to the fact that the Pringle maneuver is applied more consistently within the UGH group (88% vs. 50%, *p* = 0.027). Of interest, when comparing the median duration of the applied Pringle maneuver, no significant difference was found between the UGH and the control group (*p* = 0.417). In this context, it still seems important to emphasize that no difference in level of transaminases (AST, ALT) was observed 24 h after surgery and before discharge (*p* = 0.0381 and 0.320), with equal frequency of postoperative complications at 30 days and a trend toward a shorter hospital stay.

Several limitations of our study have to be discussed. The data are generated in a single center design and the data for laparoscopic procedures was collected retrospectively. The potential effect of a learning curve for the UGH approach throughout the study period as well as higher experience with the surgical technique of the control group are not considered. As we address the proof of principle, case numbers are small. A larger study with a prospective data collection is needed to confirm our findings.

## Conclusions

In the present study, we confirm the feasibility of a facilitated laparoscopic or robotic right hepatectomy consisting of first-hand parenchymal dissection and secondary intraparenchymal dissection of the right hepatic pedicle and right vein. Relying on the experience from living donor hepatectomies for landmark-guided parenchymal dissection, a shorter operative time is associated with a trend toward a lower complication rate and length of hospital stay. These results need to be confirmed in a prospective, randomized trial.
